# A high-resolution gridded dataset of biomass resource potentials in China with policy-aware land-use constraints for renewable energy planning

**DOI:** 10.1038/s41597-026-07689-z

**Published:** 2026-06-26

**Authors:** Li Huang, Di Fan, Ke Wang

**Affiliations:** 1 Center for Energy and Environmental Policy Research, Beijing Institute of Technology, Beijing, China; 2 Beijing Laboratory for System Engineering of Carbon Neutrality, Beijing, China; 3 Basic Science Center for Energy and Climate Change, Beijing, China, Beijing, China; 4 School of Management, Beijing Institute of Technology, Beijing, China

## Abstract

The pursuit of carbon neutrality in China demands a rapid, spatially informed scale-up of renewable energy, including biomass, yet high-resolution, policy-aware data for site-specific planning remain scarce. To bridge this gap, we develop China’s high-resolution spatially explicit biomass resource potential dataset, which integrates five biomass categories (agricultural residues, forestry residues, energy crops, animal manure, and municipal waste) at 1 km resolution for 2020, with projections to 2050. This dataset incorporates key constraints such as food security, ecological conservation, and land use suitability. It provides heat value potential distribution maps in GeoTIFF and PDF formats, and heat value potential data in Excel format. By combining multi-source geospatial data, statistical downscaling, and machine learning, this dataset enables precise assessment of resource conditions and provides forward-looking planning for biomass power deployment, rural revitalization, and carbon reduction strategies, thereby meeting China’s critical need for integrated, location-aware open data in energy and land-use decision-making.

## Background & Summary

Biomass energy ranks as the fourth largest primary energy source after coal, oil, and natural gas. It is unique among these for its ability to achieve near-zero emissions^1^; when combined with carbon capture and storage (CCS), it can even deliver negative emissions^2^. Furthermore, making full use of agricultural and forestry residues prevents air pollution from open burning and promotes a circular economy^3^, while planting perennial energy crops on degraded land can improve soil conditions and support rural development^4^. China has a vast territory, extensive arable land, and high forest coverage, harboring enormous untapped biomass potential. However, existing studies typically focus on only one or two biomass categories, lack high-resolution spatially explicit data, and insufficiently consider policy constraints such as food security and ecological protection^5–7^. This hinders a comprehensive understanding of China’s biomass resources and their effective application in energy planning.

Globally, several high-resolution biomass resource datasets have been developed, such as the EU Biomass Map and the NREL biomass resource atlas for the United States. In China, early assessments were mainly provincial or national aggregations with coarse spatial resolution, typically covering only one or two biomass categories and lacking explicit policy constraints such as food security and ecological protection^1,5^. More recent efforts improved spatial resolution but focused exclusively on energy crops^8^. Consequently, a nationwide, high-resolution (1 km), multi-category biomass dataset that simultaneously incorporates policy-aware land-use constraints and forward-looking projections has been missing. This study fills that gap by integrating five biomass types (agricultural residues, forestry residues, energy crops, animal manure, and municipal waste) with a consistent 1 km grid and by providing both a 2020 baseline and 2050 projections under the SSP2 socioeconomic pathway.

China is now the world’s largest energy consumer and CO₂ emitter. In 2022, total primary energy consumption reached 5.0 Gtce, with coal accounting for about 56% of the energy mix^9^. Despite rapid renewable energy deployment, fossil fuels still dominate. The country has committed to peak carbon emissions before 2030 and achieve carbon neutrality by 2060, which requires a dramatic increase in non-fossil energy sources, including biomass. In 2021, biomass power generation capacity was 37.9 GW, representing only a small fraction of total renewable capacity^10^, indicating a huge untapped potential. Understanding the spatial and temporal availability of biomass resources is therefore a prerequisite for effective energy transition planning.

**Fig. 1:**
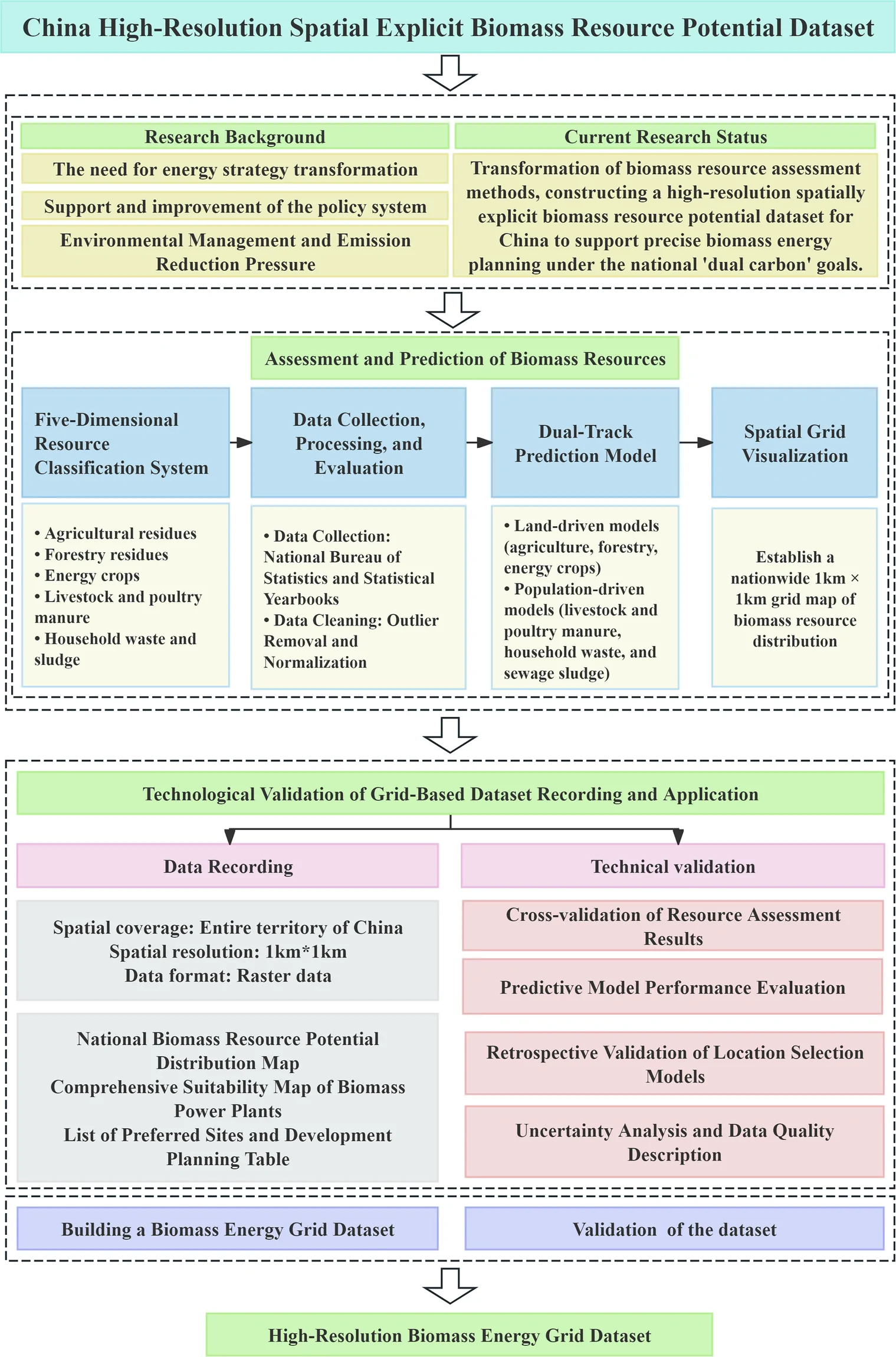
A research framework for high-resolution biomass resource potential assessment and bioenergy planning in China.

As shown in Fig. 1, to overcome these limitations, the present study introduces several methodological and conceptual innovations. First, unlike previous assessments that typically cover only one or two biomass categories, we systematically integrate five major biomass types (agricultural residues, forestry residues, energy crops, animal manure, and municipal waste) into a single, consistent high-resolution (1 km) spatial framework. Second, we explicitly incorporate policy-aware land-use constraints, including food security (protection of basic cropland), ecological conservation (avoidance of nature reserves and primary forests), and land suitability, into both the downscaling allocation and the future projection stages, which has rarely been done in existing biomass resource datasets. Third, we develop a hybrid methodological pipeline that combines statistical downscaling based on proxy variables (e.g., NPP, population density) with a random forest machine learning model trained on 20 years of historical data (2001–2020). This hybrid approach enables us to generate not only a reliable baseline map for 2020 but also forward-looking predictions for 2050 under the SSP2 socioeconomic pathway. Fourth, the resulting dataset is made publicly available in multiple formats (GeoTIFF, PDF, Excel) and has been validated against independent power plant locations and literature values, directly supporting practical applications such as biomass power plant siting and supply chain planning. Collectively, these innovations provide a spatially explicit, policy-relevant, and forward-looking data infrastructure that advances previous resource assessments in terms of resolution, completeness, and applicability to real-world energy planning.

The dataset developed in this study directly supports China’s energy transition and carbon neutrality goals. By providing spatially explicit, forward-looking biomass potential data, it enables policymakers to identify optimal locations for biomass power plants, assess the potential for coal substitution, and integrate biomass into provincial-level low-carbon development pathways. Moreover, the dataset can be used to evaluate the contribution of bioenergy to emission reduction targets under different socio-economic scenarios (e.g., SSP2), thereby helping to align renewable energy deployment with national climate commitments.

### Data sources and processing

This study establishes a comprehensive dataset encompassing multiple sources and multiple temporal and spatial scales to support the assessment and forecasting of China’s biomass resource potential, as well as the optimization of power plant siting. To ensure clarity, we distinguish three temporal categories: (1) static baseline data for 2020 (land use, NPP, population distribution) used for spatial downscaling; (2) historical time-series data for 2001–2020 (provincial statistics) used for machine learning training; and (3) future scenario data for 2050 (SSP2 land use and population) used for projections. All data have undergone standardization within a unified coordinate system (WGS 84), resolution standardization (1 km), and dimensional processing. Key data sources and processing methodologies are outlined below.

Land use data: Land use data were obtained from the 2020 remote sensing monitoring data with 1-km resolution from the RESDC of the Chinese Academy of Sciences^11^. This dataset comprises six primary categories (arable land, forest land, grassland, water bodies, construction land, and unused land) and 25 secondary categories, serving as the spatial distribution baseline for identifying biomass resources, particularly agricultural land, forest land, and subsequent marginal land. The data are freely available for academic use at http://www.resdc.cn/DOI/DOI.aspx?DOIid=54.

Population distribution data: By combining the WorldPop global 100-meter resolution population distribution raster data (available at https://www.worldpop.org/geodata/summary?id=24777)^12^ with the permanent resident data from China’s provincial statistical yearbooks^13^, cross-validation and spatial allocation are performed. The WorldPop data are distributed under CC BY 4.0 license. The combined data serve as the core proxy variable for evaluating population-driven resources such as livestock and poultry manure, domestic waste, and sludge.

Historical statistical data on biomass resources: These data were collected from official publications including the China Rural Statistical Yearbook^13^, China Forestry and Grassland Statistical Yearbook^14^, and China Environmental Statistical Yearbook^15^. All yearbooks are accessible via the National Bureau of Statistics of China (https://data.stats.gov.cn) and the EPS China Data Online platform (https://www.epsnet.com.cn) by using the search queries “Rural Statistical Yearbook”, “Forestry Statistical Yearbook”, and “Environmental Statistical Yearbook”. Annual provincial-level data for 2001–2020 were extracted. Based on these, we calculated the theoretical resource quantities and collectable potential of five types of biomass resources for each province using crop yield and residue-to-product ratios, livestock inventory and excretion coefficients, etc.

Net primary productivity (NPP) data: NPP is used as the primary spatial proxy for downscaling agricultural residues, forestry residues, and energy crops. This study uses the 2020 NPP data at 1 km resolution from the Resource and Environmental Science Data Center (RESDC) of the Chinese Academy of Sciences, available at https://www.resdc.cn. The data are derived from remote sensing inversion based on the CASA model and are distributed as annual 1 km raster files. Temporal dimension: 2020; spatial resolution: 1 km.

Future scenario data: The land use change grid data and population spatial distribution prediction dataset under the Shared Socioeconomic Pathway (SSP2)^16,17^ are used as core driving inputs for the machine learning model to predict resource potential in 2050. The SSP2 population data are available at https://zenodo.org/records/1319398, and the harmonized land use data are from the Land Use Harmonization (LUH2) project (https://luh.umd.edu).

Site selection evaluation data: By integrating the Global Biomass Power Plant Dataset from Global Energy Monitor (https://globalenergymonitor.org)^18^, national road network data, distribution and capacity data of State Grid substations, along with spatial constraint layers such as ecological protection red lines and water source protection zones, a decision-making dataset for power plant siting is constructed.

### Biomass resource classification and assessment framework

This study systematically evaluated five major biomass resources: agricultural residues, forestry residues, energy crops, animal manure, and municipal waste. The assessment follows an accounting framework that progresses from theoretical potential to harvestable potential, with the collectable potential serving as the core metric that accounts for practical losses during collection and transportation. Key evaluation parameters for each resource were synthesized from literature and industry standards. Agricultural residues refer to the literature by Gao *et al*.^19^, Dai *et al*.^20^ and Wang *et al*.^21^; Forestry residues refer to the literature by Gao *et al*.^19^ and McKendry *et al*.^22^; Energy crops refer to the research by Wang *et al*.^23^; Animal manure refers to the literature by Tian *et al*.^24^; Municipal waste refers to the literature by Gao *et al*.^19^ and the literature by Tchobanoglous *et al*.^25^. Detailed information is in Supplementary Table S1 (provided as a separate Excel file).

#### Agricultural residues

The potential assessment of agricultural residues covers the main food crops and cash crops. The theoretical resource amount is calculated based on the crop yield and the corresponding ratio of residues to products. The potential of collection further considers the loss during the process of collection, baling and transportation.

1 $${{CP}}_{{AR}}=\mathop{\sum }\limits_{i=1}^{n}{P}_{i}\times {RP}{R}_{i}\times {C}_{i}\times {{LHV}}_{{AR}}$$

${{CP}}_{{AR}}$ represents the collectable potential of agricultural residues, ${P}_{i}$ represents the yield of crop i, ${RP}{R}_{i}$ represents the residue-to-product ratio of crop i, a parameter defined as the ratio of the mass of residues, such as straw and stems, generated after crop harvesting to the mass of the economic product. ${C}_{i}$ represents the collection coefficient of residues from crop i, which reflects the proportion that cannot be recovered due to leftover and losses during collection and transportation in the field. ${{LHV}}_{{AR}}$ represents the lower heating value of agricultural residues, that is the amount of heat released when a unit mass of material is completely combusted.

### Forestry residues

The potential of forest residues is divided into three subtypes: tending residues, harvesting residues and orchard pruning residues. The potential is based on the area of different forest land and the corresponding biomass yield per unit area, and the collection coefficient is introduced to reflect the actual available resources.

2 $$C{P}_{{FR}}=\mathop{\sum }\limits_{j=1}^{n}{A}_{j}\times {Y}_{j}\times {C}_{j}\times {LH}{V}_{{FR}}$$

$C{P}_{FR}$ denotes the collectable potential of forest residues, ${A}_{j}$ represents the area corresponding to the j th forestry activity, ${Y}_{j}\,$ denotes the yield per unit area for the j th forestry activity, ${C}_{j}$ indicates the collection coefficient for the j th forest residue, and ${LH}{V}_{{FR}}$ signifies the lower heating value of forest residues.

### Animal manure

The resource amount of animal manure is estimated by the number of livestock and poultry or the number of livestock and poultry sold, combined with the daily excretion coefficient, collection coefficient and low calorific value of different livestock and poultry, and then the energy potential is calculated.

3 $$C{P}_{{AM}}=\mathop{\sum }\limits_{k=1}^{n}{N}_{k}\times {T}_{k}\times {E}_{k}\times {D}_{k}\times {C}_{k}\times {LH}{V}_{{AM}}$$

$C{P}_{AM}$ denotes the collectable potential of animal manure, ${N}_{k}$ represents the number of livestock or poultry of type *k*, ${T}_{k}$ denotes the reproductive or rearing cycle of type *k* livestock or poultry, ${E}_{k}$ indicates the daily excretion coefficient of type *k* livestock or poultry, that is the mass of fresh manure produced per animal per day, ${D}_{k}$ signifies the dry matter content of manure from type *k* livestock or poultry, ${C}_{k}$ denotes the collection coefficient for manure from livestock species *k*, and ${LH}{V}_{AM}$ represents the lower heating value of animal manure.

### Municipal waste

The amount of domestic waste and sludge is highly correlated with the population and the per capita rate of domestic waste. The potential of domestic waste is estimated based on the population data and the per capita rate of domestic waste, and the harmless treatment rate is considered.

4 $$C{P}_{{SW}}={POP}\times G\times {R}_{t}\times {LH}{V}_{{SW}}$$

$C{P}_{{SW}}$ denotes the collectable potential of municipal solid waste, POP represents the urban population, G indicates the daily per capita municipal solid waste generation, ${R}_{t}$ signifies the rate of municipal solid waste harmless treatment, and ${LH}{V}_{{SW}}$ denotes the lower heating value of municipal solid waste.

The potential of sewage sludge is estimated from chemical oxygen demand emissions and converted into energy equivalents using calorific value equivalents.

5 $$C{P}_{{SS}}={COD}\times {LH}{V}_{{COD}}$$

$C{P}_{SS}$ denotes the collectable potential of sewage sludge, COD represents the total chemical oxygen demand emissions in wastewater, and ${LH}{V}_{C{OD}}\,$ indicates the energy equivalent of COD.

### Energy crops

Given the absence of a systematic statistical yearbook for energy crops in China, quantitative assessments of their resource potential face challenges due to data scarcity. To address this issue, this study employs a method based on spatially explicit benchmark data for inversion and interpolation. The core of this approach lies in utilizing the high-spatial-resolution dataset published by Wang *et al*.^8^ as a reliable benchmark. This dataset provides the spatial distribution of biomass resource potential for five key energy crops in 2018 across two categories of marginal land: grassy crops (including Miscanthus and switchgrass) and woody crops (including willow, eucalyptus, and poplar).

The first category of marginal land was directly identified based on current land use types, primarily including shrublands, sparse woodlands, grasslands of varying coverage, lacustrine zones, tidal flats, saline-alkali lands, and bare land. Following preliminary screening, the study further excluded lands located within nature reserves, slopes steeper than 25 degrees, and areas classified as primary pastoral zones (such as the mid-to-high grasslands of Xinjiang, Qinghai, Inner Mongolia, Ningxia, and Tibet). The remaining areas were ultimately defined as Category I marginal lands. Spatially, these lands are neither cultivated fields nor primary forested areas, typically exhibiting low agricultural productivity or ecologically fragile conditions. The second category of marginal land was identified based on temporal changes in agricultural land use, specifically referring to abandoned farmland. Building on this, we assumed that the yield per unit area for each energy crop remains relatively stable in the short term, with interannual variations in resource potential primarily driven by changes in the available area of marginal land. Consequently, by constructing an area-proportion scaling model, we achieved a scientific estimation of resource potential for both historical and future years.

Specifically, the energy crops resource potential for the target year t is calculated using the following formula:

6 $${Potentia}{l}_{c,l,t}={Potentia}{l}_{c,l,2018}\times \left(\frac{{Are}{a}_{l,t}}{{Are}{a}_{l,2018}}\right)$$

${Potentia}{l}_{c,l,t}$ denotes the resource potential (in tonnes) of crop variety c on land type l for target year t. ${Potentia}{l}_{c,l,2018}$ represents the resource potential of crop variety c on land type l for the baseline year 2018, sourced directly from the publicly available dataset by Wang *et al*.^8^.

${Are}{a}_{l,t}$ denotes the area of marginal land type l in target year t. ${Are}{a}_{l,2018}$ represents the area of marginal land type l in the baseline year.

Following the determination of resource potential for each grid cell, spatial aggregation enables the derivation of total potential at provincial and national levels, calculated using the following formula:

7 $${Nationalpotentia}{l}_{t}={\varSigma }_{c}{\varSigma }_{l}{Potentia}{l}_{c,l,t}$$

In the absence of direct observational data, this method constructs a transparent, repeatable, and logically rigorous evaluation pathway by coupling high-precision baseline spatial data with dynamic land use area information. It effectively reveals the spatiotemporal dynamics of China’s energy crops resource potential, providing crucial data support for strategic planning and policy formulation in biomass energy.

This complete classification system and parameter scheme not only provide a standardized method for historical resource assessment, but more importantly, it offers clear definitions of target variables and computational logic for machine learning models, ensuring consistency and scientific rigor throughout the entire process of learning from historical data to future spatial predictions.

### Machine learning prediction model

This study constructs a machine learning model based on historical data to predict the future potential of China’s biomass resources. By integrating the available potential data and its driving factors of five types of biomass resources from 2001 to 2020, a prediction framework based on random forest is established. The model can capture the complex nonlinear relationship between biomass potential and various driving factors, and provides a reliable tool for long-term prediction.

### Feature selection

Based on the formation mechanisms of biomass resources and the data availability, we constructed a four-part feature system comprising macroeconomic characteristics, socio-demographic characteristics, resource endowment characteristics, and resource collection and waste-treatment characteristics^26^.

Macroeconomic characteristics primarily include GDP growth rate and industrial structure indicators, which indirectly influence biomass potential by affecting energy demand and agricultural productivity. Social development features cover variables such as total population, urbanization progress, and consumption patterns, directly impacting waste generation and land use changes. Resource endowment features involve natural environmental elements like arable land area, forest coverage, and marginal land resources, forming the material foundation for biomass production. Technological progress features include parameters such as collection coefficients and conversion efficiency of various biomass types, demonstrating how technological advancements enhance resource utilization rates.

Table 1 details the feature variable system and its data sources. These features collectively form the input matrix for the machine learning model, encompassing both direct factors affecting biomass potential and indirect driving elements, thereby providing a comprehensive information foundation for accurate prediction.

**Table 1:** Feature variable system and data sources.

Characteristic category	Specific variables	Unit	Data source
Macroeconomic characteristics	GDP growth rate	%	Refer to the literature by Keywan Riahi *et al*.^17^.
	The proportion of the secondary industry	%	Refer to the literature by Keywan Riahi *et al*.^17^.
Socio-demographic characteristics	Total population	people	Refer to the literature by B Jones *et al*.^16^.
	Urbanization rate	%	China Urban Statistical Yearbook^43^
	Urban population density	people/km²	China Urban Statistical Yearbook^43^
Resource endowment characteristics	Agricultural acreage	hectares	China Rural Statistical Yearbook^13^
	Woodland area	hectares	China Forestry and Grassland Statistical Yearbook^14^
	Marginal land area	hectares	Land use data measurement^17^
Resource collection and waste-treatment characteristics	The coefficient for collecting straw	/	Refer to the literature by Gao *et al*.^19^
	Collection rate of livestock and poultry manure	%	China Rural Statistical Yearbook^13^
	The rate of harmless waste treatment	%	China Urban Statistical Yearbook^43^

During the data preprocessing phase, we standardized all continuous variables to eliminate scale effects. Missing values were filled using time series or spatial interpolation methods. The resulting panel dataset, covering complete observations from 2001 to 2020 across all 31 provinces in China, provided ample samples for model training. The proportion of missing values in each input variable was generally below 5%, and detailed missing rates along with their handling methods are summarized in Supplementary Table C1. Consumption-related patterns were not included as a standalone variable because consistent provincial time-series data were unavailable; they were represented indirectly by GDP-related indicators, total population, urbanization rate, and urban population density. Derived terms used in model interpretation, such as squared terms and interaction terms, were constructed from the variables listed in Table 1. Potential multicollinearity among predictors, particularly between urbanization rate and urban population density, was assessed before model training. Both variables were retained because they capture different dimensions of urbanization: the share of population living in urban areas and the spatial concentration of urban residents.

### Construction of random forest model

Random forest is an ensemble learning algorithm, which improves the accuracy and stability of the model by constructing multiple decision trees and synthesizing their prediction results. In this study, the random forest regression algorithm is used to establish a prediction model for each type of biomass resources. An overview of the training and prediction workflow is provided in Supplementary Fig B2.

The basic principle of the model can be expressed by the following formula:

Let the input feature space be ***X***, containing p feature variables, and the output variable be biomass resource potential *Y*. For a random forest comprising N decision trees, the predicted value of the *i*-th sample can be expressed as:

8 $$Y=(1/N){\sum }_{k=1}^{N}{T}_{k}({\boldsymbol{X}})$$

*Y* denotes the final prediction output of the random forest, N represents the total number of decision trees within the forest, and ${T}_{k}({\boldsymbol{X}})$ indicates the prediction result of the kth decision tree for the input feature vector ***X***.

The model construction process comprises three critical steps: First, multiple sub-training sets are generated from the original dataset via Bootstrap sampling, providing differentiated learning samples for each decision tree. Second, during node splitting in decision trees, a feature subset (typically the square root of total features) is randomly selected as candidate features, which effectively enhances the model’s generalization ability. Finally, the prediction results from all decision trees are averaged to obtain the final output of the random forest^26–28^.

The training dataset was constructed from provincial-level panel data for 31 Chinese provinces over the period 2001–2020^29–31^. For each biomass type, we assembled a feature matrix consisting of the variables listed in Table 1 (e.g. GDP growth rate, urbanization rate, cropland area, collection coefficients, etc.) and the corresponding target variable (collectable biomass potential calculated from statistical yearbooks and literature-based parameters). After removing rows with missing target values, the final training set contained 620 samples (31 provinces × 20 years).

To evaluate model performance while respecting the temporal order, we employed time-series cross-validation with five splits (implemented via Time Series Split in scikit-learn). In each split, earlier years are used for training and later years for validation, preventing future information from leaking into the training process. To reduce overfitting, we took three complementary measures:

1. Feature selection – a preliminary random forest (100 trees, max depth = 6) was trained to rank feature importance; only the most important features were retained for the final model. The contribution of each feature to the model output is visualized using SHAP summary plots (Supplementary Fig A1a-e).
2. Regularized hyperparameters – after grid search with cross-validation, the final models used: n_estimators = 150, max_depth = 6, min_samples_leaf = 15, min_samples_split = 20, and the feature subset size set to the square root of the total number of features. These settings balance bias and variance.
3. Monitoring overfitting – we compared training and validation errors. For most biomass types, the training RMSE was 40–60% lower than the validation RMSE, indicating some remaining overfitting (common in spatial-temporal prediction with strong autocorrelation). Nevertheless, the high cross-validated R² values confirm the models’ overall predictive power. The detailed cross-validation metrics for each biomass type are reported in Supplementary Table A1. A comparison with linear regression and XGBoost (Supplementary Table A2) further confirms that random forest achieves the highest predictive robustness.

To provide a measure of uncertainty for each prediction, we used the standard deviation of predictions across all decision trees in the random forest ensemble. For a given input, each of the 150 trees produces a prediction; the standard deviation of these 150 outputs serves as an estimate of the model’s uncertainty (higher values indicate greater disagreement among trees). This uncertainty metric was computed for every sample in the training set (depending on the biomass type).

To assess potential spatial autocorrelation in model residuals, which could violate the independence assumption of conventional time-series cross-validation, we computed global Moran’s I for each year and each biomass type (Supplementary Table A3). The multi-year mean Moran’s I was close to zero for all categories (agricultural residues: −0.025; forestry residues: 0.043; energy crops: −0.059; animal manure: 0.031; municipal waste: −0.038), and the vast majority of annual p-values exceeded 0.05. This indicates that residuals are randomly distributed in space, and no additional spatial cross-validation is required. These results are provided in Supplementary Table A3.

To provide a practical measure of prediction uncertainty, we calculated for each grid cell the 90% prediction interval as the mean ±1.645 × (standard deviation across trees). Across all biomass types, the mean relative uncertainty (half-interval divided by the mean) ranged from 12% (agricultural residues) to 28% (energy crops), reflecting the higher spatial variability of marginal land distribution.

Using the trained random forest models, we projected the feature variables from 2021 to 2050 (under the SSP2 socioeconomic pathway) to obtain future trends for the five biomass categories. The input variables and assumptions used for future projections (2021–2050) are detailed in Supplementary Table C2. This predictive framework accounts for historical development patterns and incorporates scenario-based socio-economic drivers, providing a scientific basis for long-term biomass energy planning. To interpret the model predictions and identify the most influential drivers for each biomass type, we conducted SHAP (SHapley Additive exPlanations) analysis.

The SHAP summary plots for the five biomass categories are provided in Supplementary Figs A1a-A1e. For agricultural residues, cropland area and its squared term are the dominant predictors, with positive SHAP contributions, confirming that agricultural land availability is the primary determinant of residue potential. For forestry residues, forest area plays a similar dominant role. In contrast, energy crops potential is most strongly influenced by population and urbanization rate, reflecting the importance of human activity and land use pressure on marginal land. Animal manure potential is also driven by population and urbanization, while municipal waste and sludge potential is overwhelmingly determined by the GDP–population interaction, consistent with waste generation patterns. These SHAP results are fully consistent with the feature importance rankings and provide additional insight into the directionality of each factor’s effect.

### Space grid method

To integrate datasets originating from different spatial scales (pixel-based remote sensing, provincial statistics, and future scenario grids), we implemented a three-step workflow. The complete downscaling workflow is illustrated in Supplementary Fig B1.

Step 1: Spatial harmonization to a common grid. All spatial datasets were transformed to WGS 84 and resampled to a consistent 1 km × 1 km grid. For continuous variables (e.g., NPP, population density), bilinear interpolation was used. For categorical variables (e.g., land use type), nearest-neighbor resampling was applied. The 2020 land use raster was used as the master grid; all other spatial layers were masked and clipped to exactly match its extent and cell alignment.

Step 2: Disaggregation (downscaling) of provincial totals to 1 km grids. Provincial-level collectable potential for each biomass category was allocated to 1 km grid cells using spatially explicit proxy variables. The allocation assumes that within a given province, the spatial pattern of a biomass resource is proportional to the proxy variable on eligible land. Specific proxy variables and formulas are provided below for each biomass type (see Equations 9–14). This approach ensures mass balance: the sum of all grid-cell potentials within a province equals the independently calculated provincial total.

All downscaling operations were implemented in Python 3.10 using the rasterio (v1.3.6) and numpy (v1.26.4) libraries. The workflow for each biomass type consisted of four sub-steps: (i) masking – suitable land use types (e.g., cropland for agricultural residues, forest land for forestry residues, marginal land for energy crops) were extracted; (ii) weight calculation – for each grid cell within a province, the allocation weight was computed as the product of the proxy variable (NPP for agricultural/forestry/energy crops; rural population for animal manure; urban population for municipal waste) and the corresponding mask; (iii) normalization – the cell-specific weight was divided by the sum of weights over the province; (iv) allocation – the provincial total was multiplied by the normalized weight to obtain the grid-cell value, as formalized in Eqs. (9)-(14).

Step 3: Aggregation (upscaling) from grids to provinces/regions. For validation and reporting, 1 km grid values can be aggregated to any administrative unit by overlaying the corresponding boundary shapefile and summing all grid cells falling within each unit. The provincial summaries provided in the Excel files accompanying this dataset were generated using official provincial boundaries from the RESDC database.

Validation of downscaled outputs. The downscaled results were validated in two complementary ways. First, we compared the spatial patterns of the downscaled energy-crop potential with an independent high-resolution dataset^8^. The provincial-level comparison showed a strong positive correlation between the two datasets (Pearson’s r = 0.82), indicating consistent identification of high-potential regions. Second, we performed a retrospective validation using the locations of existing biomass power plants (Fig. 4). The fact that 52.7% of plants fall into “fully suitable” zones and another 86.7% are within 5 km of such zones demonstrates that the downscaled resource maps capture real-world resource distributions with sufficient accuracy for practical planning. These validations indirectly support the reliability of the downscaling assumptions (proportionality between residues and NPP, etc.) and the overall dataset.

The downscaling of provincial-level biomass potentials to 1 km grid cells relies on proxy variables that are spatially explicit and correlate with the actual distribution of biomass resources. For agricultural residues, forestry residues and energy crops, we use net primary productivity (NPP) as the primary proxy. NPP directly measures vegetation growth and biomass accumulation; the assumption is that within a given province, the spatial pattern of residues is proportional to the NPP on suitable land (cropland for agricultural residues, forest land for forestry residues, and marginal land for energy crops). For animal manure, we use rural population distribution as the proxy, based on the fact that livestock and poultry farming in China is predominantly located in rural areas; the assumption is that manure generation is proportional to the rural population density within a province. For municipal solid waste and sewage sludge, we use urban population distribution, assuming that waste generation scales with urban population density, a relationship widely documented in environmental statistics. It is important to note that these proxy variables are only used to spatially disaggregate the provincial totals, which are independently calculated from statistical yearbooks and literature-based parameters. The random forest model is trained on the provincial-level historical data (2001–2020) before any spatial allocation, thus avoiding circularity. The resulting gridded dataset therefore combines accounting-based totals with proxy-guided spatial distribution.

However, this downscaling approach has inherent limitations and potential biases. The proportionality assumption between NPP and residue generation may be violated in regions where agricultural management practices (e.g., straw retention, collection efficiency) vary significantly across space. Similarly, using rural population density as a proxy for manure generation assumes a constant per-capita excretion rate, which may not hold across different farming systems (intensive vs. backyard farming). Urban population density is also an imperfect proxy for waste generation because waste collection and treatment infrastructure differ greatly among cities. These simplifications could lead to over- or under-estimation in specific grid cells, particularly in areas with heterogeneous land use or rapid urbanization. Nevertheless, the validation against existing biomass power plant locations (Fig. 4) and the general agreement with literature-based totals (Table 2) suggest that the overall dataset is robust at national and regional scales. Future work could refine the downscaling by incorporating higher-resolution remote sensing products (e.g., crop type mapping, nighttime lights) or machine-learning-based spatial disaggregation techniques.

**Table 2:** Comparison with the evaluation of the existing literature.

Biomass category	Refer to the literature by Kang *et al*.^1^	Refer to the literature by Yang *et al*.^33^	Refer to the literature by Zhou *et al*.^5^	Refer to the literature by Liu *et al*.^34^
agricultural residues	7.1–10.4 EJ	ca. 8.3 EJ	ca. 7.6 EJ	ca. 6.1 EJ
forest residues	4.5–5.1 EJ	ca. 4.8 EJ	ca. 3.9 EJ	ca. 3.3 EJ
animal manure	5.6–6.3 EJ	ca. 5.2 EJ	—	—
municipal waste	0.31–0.83 EJ	ca. 0.4 EJ	—	—
sewage sludge	0.15–0.35 EJ	—	—	—
energy crops	0–10.1 EJ	—	—	—

Net primary productivity data are selected as the main proxy variable for spatial distribution of agricultural residues^8^. This method effectively characterizes vegetation growth status and productivity levels. The allocation process begins with NPP data masking based on land use types, ensuring rice straw is exclusively distributed in paddy fields while other dry-land crop residues are allocated to dry-land areas. Subsequently, the proportion of NPP value in each grid cell relative to the total provincial NPP is calculated to determine the allocated straw resource amount for that grid. The calculation formula is as follows:

9 $${Ag}{r}_{i,k}={A}_{k,p}\times ({NP}{P}_{i}\times {LandUs}{e}_{i,l}\times {Pro}{v}_{i,p})/{\Sigma }_{p}({NP}{P}_{i}\times {LandUs}{e}_{i,l}\times {Pro}{v}_{i,p})$$

This formula spatially allocates provincial agricultural residues statistics to 1 km grid cells. ${Ag}{r}_{i,k}$ represents the resource density ton/km² of residue type *k* in grid *i*, while ${A}_{k,p}$ is the total amount of that residue in province p. The allocation weight is based on the net primary productivity ${NP}{P}_{i}$ and land use type ${LandUs}{e}_{i,l}$ (e.g., paddy or dryland), constrained by the provincial identifier ${Pro}{v}_{i,p}$.

The spatial distribution logic of forestry residues is similar to that of agricultural residues^8^. This method primarily targets residues from commercial timber, firewood, logs, and specific forest types (e.g., timber forests and economic forests). It employs NPP data and forest land use maps for weighted allocation, with the formula expressed as:

10 $${Fore}{s}_{i,k}={F}_{k,p}\times ({NP}{P}_{i}\times {LandUs}{e}_{i,l}\times {Pro}{v}_{i,p})/{\Sigma }_{p}({NP}{P}_{i}\times {LandUs}{e}_{i,l}\times {Pro}{v}_{i,p})$$

This formula allocates forestry residues from timber forests from provincial statistics to spatial grids. ${Fore}{s}_{i,k}$ denotes the density of residue type k in grid i, and ${F}_{k,p}$ is the provincial total. Allocation is weighted by grid-level NPP, forest land use type ${LandUs}{e}_{i,l}$ (e.g., woodland), and provincial affiliation ${Pro}{v}_{i,p}$. This approach ensures that residue distribution aligns with forest productivity and land use patterns.

The spatial distribution of animal manure resources is directly correlated with livestock and poultry farming volumes. This study first calculates the total provincial manure resources based on statistical data of livestock and poultry inventory and manure pollution coefficients. Given the strong correlation between farming activities and rural population distribution, the rural population data from the Land Scan Global Population Distribution dataset is used as a spatial proxy.^8^ Calculate the allocation weight by dividing the rural population in each grid by the total rural population of the province, using the following formula:

11 $${Manur}{e}_{i,p}={TManur}{e}_{p}\times ({RuralPo}{p}_{i}/{\Sigma }_{i}{RuralPo}{p}_{i})$$

${Manur}{e}_{i,p}$ denotes the manure resource quantity in grid i for province p, ${TManur}{e}_{p}$ represents the total manure resource volume for province p, and ${RuralPo}{p}_{i}\,$ indicates the rural population size in grid i.

The generation of domestic waste and sludge is closely related to the urban population size and urbanization level. Therefore, this study uses the urban population data from the Land Scan dataset as a spatial allocation proxy^8^. First, based on provincial-level statistics of domestic waste collection volumes or sludge production, the data is allocated to grid units using urban population weights. The domestic waste distribution formula is:

12 $${MS}{W}_{i,p}={TMS}{W}_{p}\times ({UrbanPo}{p}_{i}/{\Sigma }_{i}{UrbanPo}{p}_{i})$$

${MS}{W}_{i,p}$ denotes the potential resource volume of municipal solid waste in grid i for province p, ${TMS}{W}_{p}$ represents the total volume of municipal solid waste collected in province p, and ${UrbanPo}{p}_{i}$ indicates the urban population count in grid i.

The same principle is applied to the spatial distribution of sludge resources, with the formula being:

13 $${Sludg}{e}_{i,p}={TSludg}{e}_{p}\times ({UrbanPo}{p}_{i}/{\Sigma }_{i}{UrbanPo}{p}_{i})$$

The allocation logic for sewage sludge is similar to that for municipal solid waste, also using urban population as a spatial proxy. ${Sludg}{e}_{i,p}$ represents the sludge resource quantity in grid i for province p, and ${TSludg}{e}_{p}$ is the provincial total. ${UrbanPo}{p}_{i}$ the urban population in the grid. This method assumes that sludge production is related to wastewater treatment scale and urban population density, suitable for urban areas with well−developed sewage systems.

The spatial distribution of energy crops using a down-scaling method similar to that of agricultural residues^8^. The allocation process is based on the total annual energy crops yields predicted at the provincial level, which serve as the statistical foundation. A two-tier proxy indicator system is employed: First, the spatial distribution map of the two marginal land types identified in this study serves as a primary spatial constraint to ensure energy crops are only distributed on suitable marginal land. Second, net primary productivity data is used as a secondary weighted indicator to reflect potential productivity differences across various marginal land units. The spatial allocation formula is as follows:

14 $${Cro}{p}_{i,c,t}={TCro}{p}_{c,p,t}\times ({NP}{P}_{i}\times {MarginalLan}{d}_{i,l})/{\Sigma }_{p}({NP}{P}_{i}\times {MarginalLan}{d}_{i,l})$$

${Cro}{p}_{i,c,t}$ denotes the resource density of energy crop *c* within grid cell $\,{i}$ during target year t. ${TCro}{p}_{c,p,t}$ represents the total yield of energy crop *c* in province p during the same year. ${NP}{P}_{i}$ indicates the net primary productivity value for grid cell i. ${MarginalLan}{d}_{i,l}$ signifies whether grid cell i belongs to marginal land type l.

Finally, through the above process, a comprehensive biomass resource grid dataset with high spatial resolution was generated, covering agricultural residues, forestry residues, energy crops, animal manure, domestic waste, and sludge. This dataset provides a precise data foundation for subsequent spatial analysis, potential assessment, and planning.

## Data Records

The datasets generated in this study are publicly available on figshare (https://doi.org/10.6084/m9.figshare.30993439)^32^. The repository provides the high-resolution gridded biomass resource potential dataset in GeoTIFF, PDF, and Excel formats.

We provide datasets in three distinct formats for multidisciplinary researchers. The first format consists of biomass resource potential maps stored as GeoTIFF files (.tif) with WGS 84 projection and approximately 1 km resolution. This includes two agricultural residues maps, two forestry residues maps, two energy crops maps, two animal manure maps, two municipal solid waste and sewage sludge maps, and two integrated bioenergy potential maps, covering the distribution of five biomass types for both 2020 and 2050. The second format of the dataset is PDF files containing the calorific density distribution of the five biomass resources, measured in gigajoules per square kilometer (GJ/km²), as shown below. The third format is Excel files providing provincial-level aggregated summaries of the high-resolution maps mentioned above. This dataset can be used in numerous sub-national integrated assessment models. All files are accessible via figshare^32^.

## Data Overview

Comparing biomass resource potential data between 2020 and 2050 reveals a pronounced growth trend and structural optimization in China’s biomass energy development. In terms of total volume, the annual calorific value potential of biomass resources increased from 30.3 EJ in 2020 to approximately 48.3 EJ in 2050, representing a 59.4% growth. This upward trajectory fully reflects China’s sustained investment and development potential in the biomass energy sector. Analyzing changes in resource structure: - The calorific value of agricultural residues increased from 10.7 EJ to 16.7 EJ, a 56.1% rise, maintaining a relatively stable share of total potential; The calorific value of forestry residues will rise from 6.5 EJ to 12.7 EJ, a 95.4% increase, with a significantly higher share; the calorific value of energy crops will surge from 6.8 EJ to 9.4 EJ, a 38.1% increase; the calorific value of animal manure will increase from 5.5 EJ to 8.6 EJ, a 68.5% rise; The calorific value potential of municipal solid waste and sludge remains largely unchanged. This structural shift reflects China’s rapid expansion in energy crops cultivation and livestock farming scale, while also indicating substantial room for improvement in the resource utilization of urban and rural organic waste. Regionally, southern provinces like Yunnan, Guangxi, and Guangdong showed particularly notable growth in forestry and energy crops, while traditional agricultural powerhouses such as Henan, Shandong, and Heilongjiang maintained their leading positions in agricultural residues. This growth pattern aligns closely with China’s agricultural modernization process, forestry development strategy, and energy structure adjustment direction, providing crucial resource support for building a clean, low-carbon, secure, and efficient energy system^2,33^.

**Fig. 2:**
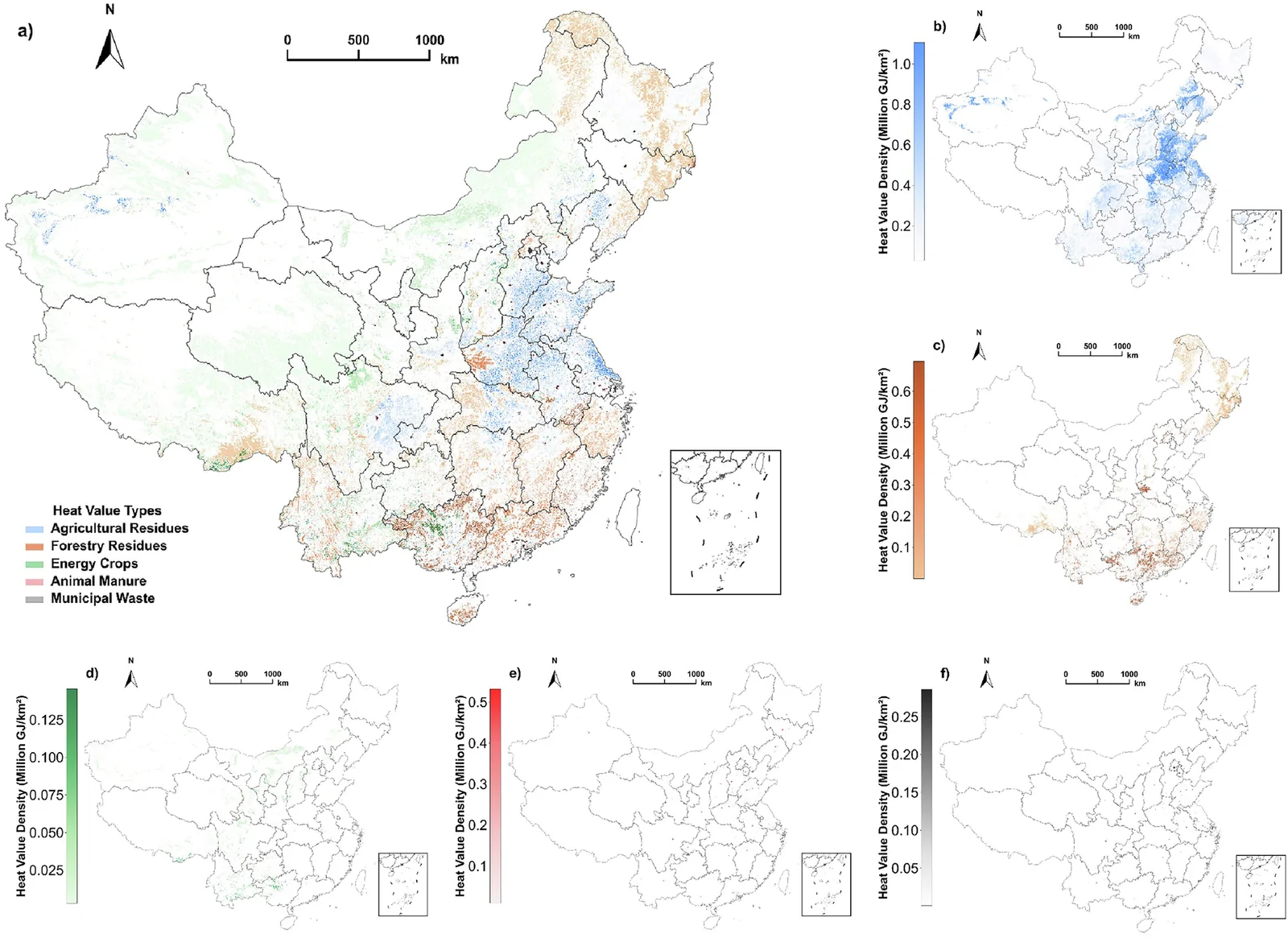
Spatial distribution of biomass calorific values in China. (**a**) shows the combined distribution of five types of calorific values (agricultural residues, forestry residues, energy crops, animal manure, and municipal waste & sludge) in 2020. (**b**–**f**) respectively show the distribution of agricultural residues, forestry residues, energy crops, animal manure, and municipal waste & sludge.

The spatial overlay distribution map of China’s five biomass calorific value types in 2020 (Fig. 2a), derived from this study, reveals a distinct “three-tiered” spatial distribution pattern that closely aligns with the country’s topographical terraces^34^. The first tier (eastern plains and hilly regions) exhibits the highest calorific density, primarily distributed across the Northeast Plain, North China Plain, middle and lower reaches of the Yangtze River Plain, and the southeast coastal areas. Calorific density generally exceeds 500,000 GJ/km², with core areas such as the North China Plain, Yangtze River Delta, and Pearl River Delta reaching densities above 1,000,000 GJ/km². The second tier (central plateau and mountainous regions) exhibits moderate thermal energy density, encompassing the Loess Plateau, Yunnan-Guizhou Plateau, and Sichuan Basin, with densities ranging between 200,000 and 500,000 GJ/km². The third tier (western plateau and mountainous regions) possesses the lowest thermal energy density, primarily comprising the Qinghai-Tibet Plateau, Inner Mongolia Plateau, and arid northwest regions, where densities are mostly below 100,000 GJ/km².

As shown in Fig. 2b, the distribution of agricultural residues calorific value exhibits a “two belts and one area” pattern. The Northeast-North China-Middle and Lower Yangtze River Plains form the primary agricultural calorific zone. This region features contiguous cultivated land, high crop rotation indices, and abundant straw resources. Within it, the Songnen Plain, Huanghuaihai Plain, and Middle and Lower Yangtze River Plain constitute three core high-value areas, with calorific densities exceeding 800,000 GJ/km². The Sichuan Basin constitutes a high-value agricultural calorific zone in the west. Although relatively small in area, it features intensive cultivation and high calorific output per unit area. Additionally, irrigated agricultural areas such as the Hetao Plain, Guanzhong Plain, and Hexi Corridor have formed localized high-value calorific points^35^.

Figure 2c reveals that forestry thermal energy distribution aligns closely with China’s major forest regions, forming three major thermal energy clusters: Northeast-Southwest-Southeast^36^. The Northeast Forest Region, centered on the Greater Khingan Range, Lesser Khingan Range, and Changbai Mountain forest areas, features high forest coverage and substantial timber reserves, with thermal energy densities ranging from 300,000 to 600,000 GJ/km². The Southwest Forest Region primarily spans the Hengduan Mountains and Yunnan-Guizhou Plateau, characterized by rich biodiversity. Its calorific value density exhibits significant spatial heterogeneity due to complex topography. The Southeast Forest region covers the Jiangnan Hills, Nanling Mountains, and Wuyi Mountains, dominated by plantation and economic forests, with relatively uniform calorific value density.

Analysis of Fig. 2d reveals that the distribution of energy crops calorific values strictly adheres to ecological constraints and land suitability principles^37^. In the Northeast, energy crops are mainly distributed along forest edges and in some afforested areas; in the Southwest, they are concentrated on marginal lands in low mountains and hills; in the South, they overlap with some economic forests and shrublands. Energy crops cultivation avoids all nature reserves, resulting in a distinct “avoidance pattern” in caloric value distribution. This creates a fragmented and dispersed spatial pattern, preventing conflicts with ecologically sensitive areas.

Observing Fig. 2e reveals that the distribution of livestock manure calorific value is highly correlated with China’s livestock industry layout and population density, exhibiting a pattern of “concentration in agricultural areas and dispersion in pastoral areas”^38^. The highest calorific value density occurs in large-scale farming regions like the North China Plain and Northeast Plain, closely linked to the intensive pig and poultry farming in these areas. Although southern agricultural regions primarily rely on small-scale farming, their high breeding density creates contiguous areas of moderate calorific value. Traditional pastoral areas like the Inner Mongolia grasslands and the Qinghai-Tibet Plateau exhibit generally low calorific value density due to extensive grazing practices and difficulties in manure collection.

Figure 2f reveals that the distribution of calorific value from municipal solid waste and sludge strongly follows a “point-axis” pattern, closely aligning with urbanization levels and population distribution^39^. The Beijing-Tianjin-Hebei, Yangtze River Delta, and Pearl River Delta megacities form core high-calorific zones exceeding 200,000 GJ/km². Provincial capitals and prefecture-level cities create regional calorific hotspots connected by transportation corridors. Rural areas, characterized by dispersed populations and inadequate waste collection systems, exhibit generally low calorific density, forming extensive low-value background zones.

Distinct thermal value combinations characterize different regions: Northeast China relies primarily on agricultural residues and forestry thermal values, forming an “agricultural-forestry composite” type; The North China Plain relies on agricultural residues and livestock manure, forming an “agricultural-pastoral composite” pattern; The southeastern coastal region exhibits abundant thermal values across all five categories, presenting a “diverse and balanced” pattern; The southwest region is dominated by forestry and energy crops, reflecting a “forestry-energy dominant” pattern; The northwest region has relatively low thermal values across all categories, classified as a “resource-constrained” pattern.

**Fig. 3:**
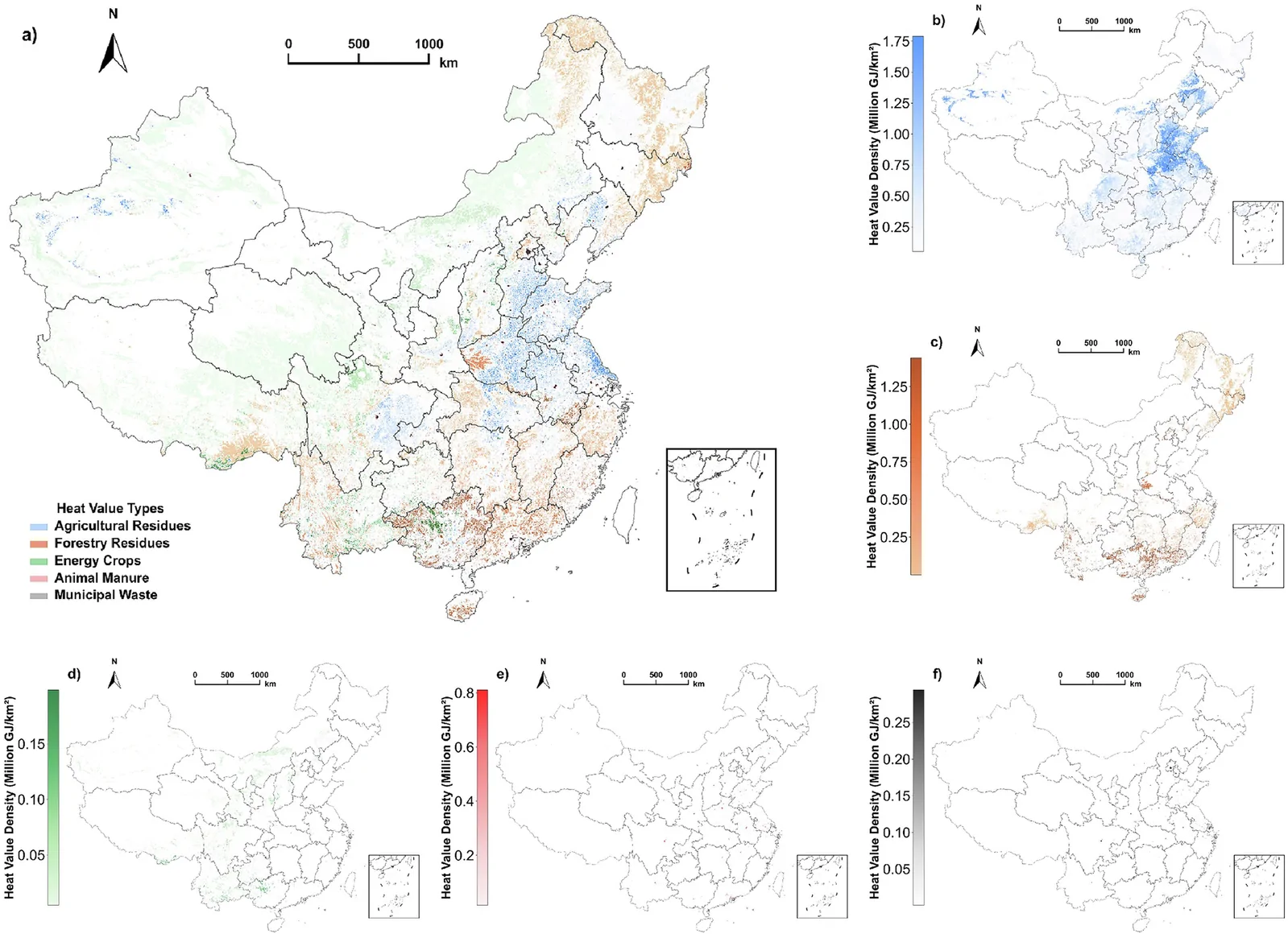
Projected spatial distribution of biomass calorific values in China for 2050. (**a**) shows the combined distribution of five types of calorific values. (**b**–**f**) respectively show the distribution of agricultural residues, energy crops, forestry residues, animal manure, and municipal waste & sludge.

Figure 3a shows that the spatial distribution of biomass calorific value in China continues to exhibit a distinct “three-tiered” pattern, closely aligning with the elevation of topographical terraces. The first tier (eastern plains and hilly regions) exhibits the highest calorific value density, generally exceeding 1 million GJ/km² across the Northeast Plain, North China Plain, middle and lower reaches of the Yangtze River Plain, and the southeast coastal areas. Key regions such as the core North China Plain, Yangtze River Delta, and Pearl River Delta achieve densities surpassing 3 million GJ/km². The second tier (central plateau and mountainous regions) exhibits moderate thermal energy density, ranging between 500,000 and 1,000,000 GJ/km² across the Loess Plateau, Yunnan-Guizhou Plateau, and Sichuan Basin. The third tier (western plateau and mountainous regions) exhibits the lowest thermal energy density, with values below 200,000 GJ/km² in the Qinghai-Tibet Plateau, Inner Mongolia Plateau, and arid northwest regions. Compared to 2020, all regions will see significant increases in thermal energy density by 2050, with the potential of biomass resources being fully tapped.

Figure 3b shows that the spatial pattern of agricultural residues calorific density in 2050 remains largely consistent with that of 2020. High-value zones exceeding 1 million GJ/km² persistently cluster across the Northeast Plain and North China Plain, while the middle and lower reaches of the Yangtze River Plain form a contiguous sub-high-value zone. This pattern’s persistence and intensification stem from historical cropping patterns. Concurrently, macroeconomic growth and the evolution of the secondary industry’s share have sustained agricultural investment and intensification. Anticipated technological advancements in straw collection coefficients are expected to directly reduce field losses, enabling more efficient conversion of theoretical resources into accessible energy potential. This will achieve a widespread increase in calorific density while maintaining stable arable land area.

Figure 3d reveals a distinct “dense in the north, sparse in the south” pattern in the distribution of energy crops calorific values by 2050. Areas with energy crops calorific values exceeding 150,000 GJ/km² are concentrated in Northeast China, the marginal land belt of Northwest China, and the middle and lower reaches of the Yellow River basin, while only scattered occurrences are found in the hilly and mountainous regions of southern China. This pattern not only reflects the dependence of energy crops development on marginal land resources but also highlights the critical role of technological progress in enhancing the utilization efficiency of marginal lands. Among the two types of marginal lands evaluated in this study, abandoned farmlands (Category II marginal lands) have historically remained underutilized due to constraints imposed by existing technological conditions. Advancements in agricultural production techniques and land reclamation capabilities—including saline-alkali soil improvement, degraded grassland restoration, and abandoned farmland reclamation—have transformed vast areas of Category II marginal land in northern and northwestern China into viable resources suitable for energy crops cultivation. This technology-driven expansion of usable marginal land significantly enhances the calorific potential of energy crops. Concurrently, population growth and urbanization have indirectly promoted energy crops cultivation on abandoned farmland by exerting pressure on arable land conservation. Overall, driven by both the increase in marginal land available for energy crops and technological advancements, the calorific potential of energy crops has been substantially enhanced, providing a crucial resource foundation for China’s biomass energy development.

Figure 3e shows that the distribution of animal manure calorific value is highly coupled with the layout of China’s large-scale livestock industry, forming high-potential dense zones centered on the North China agricultural region and the Sichuan Basin. These areas are not only major grain-producing regions but also adjacent to key consumer markets, resulting in high livestock density and intensive farming practices. Changes in population and urban population density determine meat consumption demand and the locational choice of livestock farming. The enormous resource potential in these areas stems directly from the substantial biomass generated by vast livestock populations. Additionally, improving standardized manure collection rates and processing efficiency is key to transforming the scale advantages of these farming regions into stable energy supply—a primary driver behind the significant resource growth observed in the aforementioned high-potential zones.

Figure 3c shows that high-potential zones for forest residues calorific value distribution fully overlap with China’s major forest regions, concentrated in the Greater and Lesser Khingan Ranges and Changbai Mountain forest areas in the northeast, the Hengduan Mountains in the southwest, and the southern hilly regions. This clearly indicates that forest residue potential is fundamentally constrained by the baseline and distribution of forest resources. As traditional forestry bases, the future growth of exploitable resources like thinning residues and logging residues in these regions depends not only on stable forest area but also on macro-level GDP growth. Economic development influences potential impacts on forestry investment and management intensity, driving improvements in forest management practices. Together, these factors determine the proportion and total volume of exploitable residues generated during thinning and logging operations.

Figure 3f clearly illustrates the spatial distribution of calorific density in China’s municipal solid waste and sludge. Its highly concentrated “point-axis” pattern vividly depicts the geographic patterns of population and economic activity. Major urban agglomerations such as the Yangtze River Delta, Pearl River Delta, and Beijing-Tianjin-Hebei region have formed distinct high-density calorific cores. The direct drivers of this distribution pattern are population size, urbanization levels, and population density, which collectively determine the baseline volume of waste generation. However, the key to transforming this environmental burden into a stable energy resource hinges on technological advancement, particularly improvements in the efficiency of waste harmless treatment and resource recovery. Advances in treatment technologies directly increase the proportion of waste that can be effectively collected and channeled into energy conversion processes. Notably, while future population declines may reduce absolute waste generation, continuous improvements in treatment technologies largely offset this reduction. Consequently, the calorific potential of municipal solid waste and sludge remains largely unchanged by 2050 compared to 2020. This underscores the pivotal role of technological innovation in unlocking the potential of existing resources and achieving sustainable resource conversion.

Comprehensive analysis indicates that by 2050, the spatial patterns of China’s five biomass resource categories will largely persist, though their scales will generally increase. Agricultural and forestry residues remain predominant with pronounced concentration; energy crops potential is gradually unlocked on marginal lands; while animal manure and urban waste resources are deeply coupled with population and economic distribution. This spatial pattern provides crucial guidance for regional biomass energy development and industrial chain planning. Regionally, the spatial distribution of biomass calorific value resources has undergone significant changes. While eastern coastal regions maintain high resource density, their relative share has declined. Central and western regions, particularly southwestern provinces, have seen a significant increase in resource share, fostering a more balanced regional development pattern. Eastern regions, while maintaining efficient utilization of municipal solid waste and sludge, have prioritized the development of forestry residues and energy crops, reflecting an upgrade in resource development quality. Central regions, as major producers of agricultural residues and animal manure, have achieved stable resource growth through technological advancement. Leveraging abundant land resources and ecological advantages, the western region has achieved breakthroughs in energy crops and forestry residues, emerging as a key growth pole for biomass resource development.

The trajectory from 2020 to 2050 fully reflects the profound influence of national energy strategies and policy orientations^39^. The introduction of carbon neutrality goals, the formulation of renewable energy development plans, and the advancement of agricultural and rural modernization collectively shape the policy environment for biomass resource development^40^. Looking ahead, with further technological breakthroughs and continuous policy refinement, China’s biomass calorific resources will maintain steady growth. Resource structures will become more optimized, spatial layouts more rational, providing robust support for building a clean, low-carbon, secure, and efficient energy system. Concurrently, key attention must be given to balancing resource development with ecological conservation, synergizing technological innovation with industrial advancement, and unifying regional coordination with efficiency enhancement to achieve sustainable development of biomass energy^41,42^.

The spatial patterns revealed in Fig. 2 and Fig. 3 provide actionable insights for China’s biomass energy strategy. First, the persistent “three-tiered” pattern (high density in eastern plains, moderate in central plateaus, low in western highlands) suggests that near-term bioenergy infrastructure should be prioritized in the Northeast Plain, North China Plain, and the middle-lower Yangtze River Plain, where resource density exceeds 1 million GJ/km². These areas are suitable for large-scale biomass power plants and integrated collection-storage-transportation hubs. Second, the rapid growth of forestry residues and energy crops in southern provinces (Yunnan, Guangxi, Guangdong) indicates that decentralized, small-to-medium scale gasification or combined heat and power (CHP) facilities could be more appropriate in these mountainous regions, reducing transportation costs. Third, the strong spatial coupling between animal manure and agricultural residues in the North China Plain calls for co-digestion plants that process multiple feedstock types, improving plant utilization rates and reducing seasonal supply fluctuations. Fourth, the “point-axis” pattern of municipal waste and sludge (highly concentrated in megacities) suggests that waste-to-energy plants should be sited within or near major urban agglomerations, where waste generation is abundant and district heating networks exist. Fifth, the expansion of energy crops on marginal land in northern and northwestern China implies that bioenergy development can support land restoration and rural revitalization, but careful logistics planning is needed because these areas are often remote. Overall, the dataset enables multi-criteria decision-making: policymakers can overlay resource maps with transmission grid, road network, and protected area layers to identify optimal zones for different types of bioenergy projects, thereby aligning renewable energy targets with ecological and socio-economic goals.

## Technical Validation

To demonstrate the reliability and validity of this dataset (China’s high-resolution spatially explicit biomass resource potential dataset), we conducted validation at two levels: First, we performed retrospective validation of the derived comprehensive suitability maps using the distribution of existing biomass power plants. Second, we compared the resource potential assessment results from this study with existing literature to illustrate the reasonableness of their magnitude.

### Suitability evaluation and validation based on existing power plant layout

This paper uses the gridded biomass resource potential dataset to generate a comprehensive suitability map for biomass power plants by coupling infrastructure, economic cost, and environmental constraints.

**Fig. 4:**
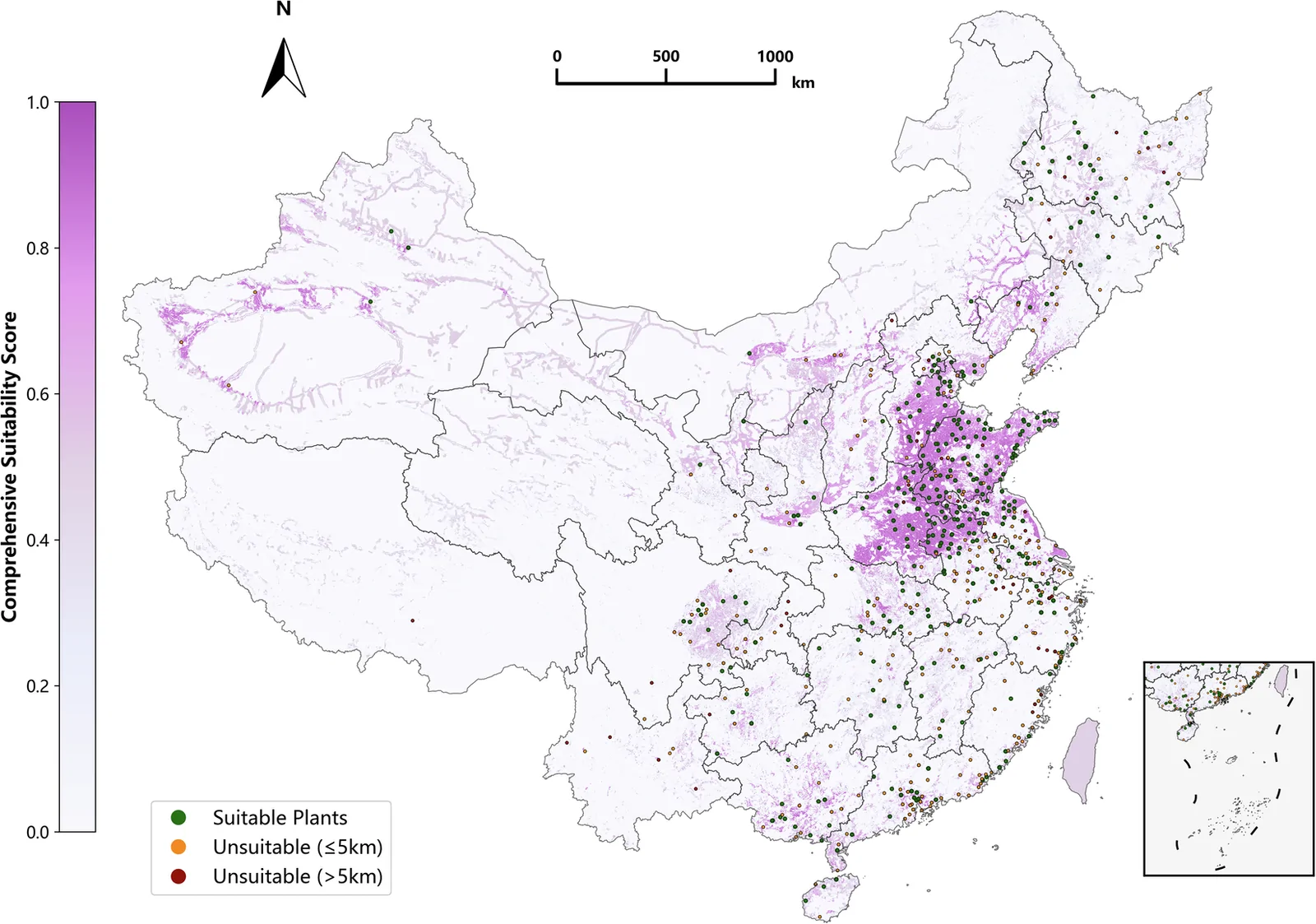
Suitability assessment of biomass power plant.

The analysis results (Fig. 4) indicate that 518 power plants are located in the “fully suitable” zones defined by the comprehensive adaptability map, accounting for 52.7% of the total. This directly demonstrates the high consistency between the spatial optimization site selection scheme based on this dataset and the successful project layouts in reality, highlighting the guiding value of the dataset in practical applications.

Notably, among the remaining 465 “unsuitable” power plants, 403 (86.7% of the unsuitable plants) are within 5 kilometers of the “fully suitable” zone boundary. This suggests that most “unsuitable” plants are not fundamentally miscalculated but rather situated at the edge of optimal zones. These plants can compensate for minor location disadvantages through fine-tuning of procurement, storage, and transportation systems. Their existence reflects historical influences of non-technical factors in actual projects, such as land acquisition and community acceptance. This finding indirectly confirms that the dataset’s spatial resolution is sufficiently refined to capture subtle differences in resource endowments and location conditions.

Only 62 (13.3%) power plants are located outside optimal zones. Most of these projects were constructed during the early stages of biomass energy development, constrained by technological approaches, environmental standards, or grid infrastructure at the time, resulting in deviations from the current optimal locations assessed through comprehensive data analysis. Collectively, the 52.7% fully suitable ratio and the fact that 86.7% of unsuitable plants are within 5 km of fully suitable zones strongly validate the rationality and practicality of this resource dataset in supporting scientific siting for biomass power plants.

### Comparison with existing literature

To validate the dataset’s reliability in total resource assessment, we compared its 2020 biomass calorific potential results with representative prior studies (Table 2). These comparisons with independent, previously published resource inventories confirm the reliability of our dataset across different biomass categories, demonstrating its superior comprehensiveness and accuracy.

Regarding agricultural residues, the calorific potential assessed in this study was 10.67 EJ, consistent with the findings reported by Kang *et al*.^1^, Yang *et al*.^33^ and Zhou *et al*.^5^. Our study has included a broader range of crop types and utilized more recent yield data, accurately capturing agricultural production growth. Regarding forestry residues, the study’s evaluation result of 6.5 EJ is significantly higher than most early studies, such as Kang *et al*.^1^ and Liu *et al*.^34^. The totals stand at approximately 3.3 EJ. This discrepancy primarily stems from the study’s integration of more comprehensive forest resource inventories, advanced remote sensing inversion techniques, and refined biomass scaling factors, which more fully revealed the potential supply of forestry residues. For energy crops, the marginal land assessment yielded 6.80 EJ, lower than that reported by Liu *et al*.^34^, but it completely falls within the range of Kang *et al*.^1^. Our evaluation adopted a more conservative approach by strictly adhering to sustainability constraints such as ecological protection redlines, avoiding development in ecologically sensitive areas, which demonstrates the rigor of the assessment framework. The evaluation results for animal manure align with the findings reported by Kang *et al*.^1^ and Yang *et al*.^33^. The results showed high consistency, validating the accuracy of this study in livestock inventory calculation and pollutant parameter selection. The combined assessment of domestic waste and sludge yielded 0.90 EJ, consistent with the findings reported by Kang *et al*.^1^. The sum of the upper limits of municipal solid waste and sewage sludge is similar, which is consistent with the increasing trend of waste production with the advancement of urbanization.

Overall, the assessment results are broadly consistent with previous national and provincial estimates, although several categories lie near the upper bounds or are slightly higher than earlier reported values. These differences can be attributed to the use of updated statistical records, a refined 1 km spatial framework, and more comprehensive resource coverage. For example, the estimated agricultural-residue potential of 10.67 EJ is close to the upper range reported by Kang *et al*. (7.1–10.4 EJ), while the estimated energy-crop potential of 6.80 EJ remains within the broad range reported by Kang *et al*. (0–10.1 EJ). The higher forestry-residue estimate reflects the integration of more comprehensive forest resource inventories, remote-sensing-based allocation, and refined biomass scaling factors. These comparisons support the reliability of the dataset while clarifying why some estimates differ from earlier studies.

## Usage Notes

The “China’s high-resolution spatially explicit biomass resource potential dataset” constructed in this paper provides comprehensive and detailed spatial data support for the precise planning and scientific decision-making of biomass energy in China. To ensure users can fully and correctly utilize this dataset, the following explanations are provided. Regarding data content and structure, the core strengths of this dataset lie in its comprehensive spatiotemporal coverage and systematic resource classification. Covering both the 2020 baseline year and the 2050 future projection, the data supports full-cycle research from current status analysis to long-term strategic planning. Spatially, it employs a 1 km × 1 km high-resolution grid format, enabling clear visualization of intrinsic patterns and spatial heterogeneity in biomass resource distribution. This dataset systematically integrates five major biomass resources: agricultural residues, forestry residues, energy crops, animal manure, and municipal solid waste and sludge. To meet diverse user needs, data is provided in three formats, GeoTIFF, PDF, and Excel, suitable for GIS spatial analysis, comprehensive evaluation model inputs, and macro-level statistical queries, respectively.

This dataset demonstrates broad applicability across potential scenarios: First, by identifying resource-rich zones, it provides quantitative evidence for optimizing macro-level biomass energy planning and developing provincial roadmaps, supporting national and regional energy strategy formulation. Second, as a core input for project-level decision-making, it directly supports systematic site selection planning for biomass power plants and biorefineries, further optimizing integrated collection, storage, and transportation supply chain systems. Finally, it provides high-quality spatially explicit data support for multidisciplinary research in energy geography and resource-environment economics.

To maximize the utility of this dataset, we propose the following recommendations: First, users should clearly define research objectives to select appropriate data subsets. It is recommended to directly utilize the generated “Five-Heat-Value-Type Overlay Distribution Map” or refer to the site optimization logic in the literature to avoid potential overestimation caused by simple overlaying of underlying classification maps. Simultaneously, users must recognize and address the inherent limitations of the dataset: final accuracy depends on the precision and timeliness of input source data (e.g., land use, population distribution). For future projections, users should understand that this work is based on the SSP2 socioeconomic pathway and machine learning models. Actual developments may deviate due to policy adjustments, technological breakthroughs, or market fluctuations. Therefore, it is strongly recommended that users—especially when making precise decisions at the micro-level—cross-validate findings with the latest local statistical data and field investigations to ensure decision robustness.

## Supplementary information

Supplementary Information

Supplementary Information

## Data Availability

The datasets are available at figshare: https://doi.org/10.6084/m9.figshare.30993439.

## Code availability

The code used for calculating agricultural, forestry residues, and energy crops is written in Python and available from https://github.com/spothuang1105-ai/-High-resolution-gridded-biomass-resource-potentials-in-China.
