# Supplementary material for: A high-resolution gridded dataset of biomass resource potentials in China with policy-aware land-use constraints for renewable energy planning
Source: Sci Data. 2026 Jun 26;13:1430. doi: 10.1038/s41597-026-07689-z (PMC13672364; doi:10.1038/s41597-026-07689-z)
Supplement: Supplementary file 1 [file 41597_2026_7689_MOESM1_ESM.docx]

# Supplementary material

## A. Uncertainty analysis data

Table A1 summarizes the time‑series cross‑validation performance of the random forest model for the five biomass types. Metrics include root mean square error (RMSE), mean absolute error (MAE), coefficient of determination (R²), training RMSE, and overfitting percentage. R² values range from 0.79 to 0.93, indicating high explanatory power. Agricultural residue achieves the highest R² (0.93), while forestry residue exhibits a slightly lower R² (0.79), likely due to stronger spatial heterogeneity of forestry data. The overfitting percentages (negative values indicate training error lower than validation error) are all small or negative, demonstrating that regularization measures (e.g., feature selection, limited tree depth, and minimum leaf samples) effectively prevent overfitting. This table confirms that the random forest model reliably captures the spatiotemporal variations of biomass calorific values.

Table A1. Random forest model performance (cross-validated)

| **Biomass type** | **RMSE**  **(mean)** | **RMSE**  **(std)** | **MAE**  **(mean)** | **MAE**  **(std)** | **R²**  **(mean)** | **R²**  **(std)** | **Train RMSE** | **Overfitting**  **(%)** |
| --- | --- | --- | --- | --- | --- | --- | --- | --- |
| Agricultural residue | 59.25 | 36.15 | 34.75 | 19.93 | 0.93 | 0.04 | 33.05 | -44.22 |
| Animal manure | 33.25 | 23.77 | 22.40 | 15.50 | 0.83 | 0.18 | 18.94 | -43.06 |
| Forestry residue | 70.40 | 67.91 | 33.46 | 25.92 | 0.79 | 0.19 | 53.28 | -24.33 |
| Municipal waste & sludge | 6.66 | 5.51 | 2.79 | 2.10 | 0.80 | 0.20 | 4.89 | -26.56 |
| Energy crop | 86.30 | 40.10 | 47.73 | 24.22 | 0.83 | 0.13 | 91.13 | 5.59 |

Table A2 compares the predictive performance of three models – random forest, linear regression, and XGBoost – across the five biomass types. Linear regression yields extremely large RMSE and negative R² values for animal manure, forestry residue, and municipal waste & sludge, indicating its inability to capture non‑linear relationships. XGBoost performs comparably to random forest, but random forest achieves high average R² (0.79–0.93) and low RMSE across all categories. For example, random forest gives a slightly higher R² (0.93) than XGBoost (0.92) for agricultural residue, and a higher R² (0.83 vs. 0.80) for energy crop. Although XGBoost shows a marginally higher R² (0.89) than random forest (0.83) for animal manure, random forest outperforms or ties with XGBoost on the other four types. Overall, random forest demonstrates the best robustness and generalization ability and is therefore selected as the primary model for this study.

Table A2. Comparison with linear regression and XGBoost

| **Biomass type** | **Model** | **RMSE_mean** | **RMSE_std** | **MAE_mean** | **MAE_std** | **R2_mean** | **R2_std** |
| --- | --- | --- | --- | --- | --- | --- | --- |
| Agricultural residue | Linear regression | 60.41 | 70.41 | 48.21 | 62.48 | 0.86 | 0.20 |
| Agricultural residue | XGBoost | 45.40 | 19.73 | 29.86 | 14.95 | 0.92 | 0.10 |
| Animal manure | Linear regression | 9.85E+12 | 1.97E+13 | 9.71E+12 | 1.94E+13 | -1.83E+23 | 3.65E+23 |
| Animal manure | XGBoost | 26.74 | 15.57 | 17.47 | 9.79 | 0.89 | 0.10 |
| Forestry residue | Linear regression | 180.02 | 230.93 | 152.66 | 200.99 | -6.02 | 12.40 |
| Forestry residue | XGBoost | 52.03 | 48.39 | 23.59 | 16.15 | 0.86 | 0.10 |
| Municipal waste & sludge | Linear regression | 1.92E+12 | 3.84E+12 | 1.89E+12 | 3.78E+12 | -6.94E+23 | 1.39E+24 |
| Municipal waste & sludge | XGBoost | 4.88 | 5.22 | 2.16 | 2.21 | 0.89 | 0.13 |
| Energy crop | Linear regression | 1182.40 | 491.42 | 979.62 | 429.93 | -34.37 | 31.47 |
| Energy crop | XGBoost | 84.23 | 52.08 | 55.58 | 40.57 | 0.80 | 0.22 |

Table A3 presents the global Moran’s I and corresponding p‑values of model residuals for three biomass types (agricultural residue, forestry residue, and energy crop) over the period 2001–2020. For most years, the p‑values exceed 0.05, and the multi‑year mean Moran’s I is close to zero (agricultural residue: -0.025; forestry residue: 0.043; energy crop: -0.059). Although a few individual years (e.g., 2011 for agricultural residue) exhibit significant Moran’s I values, the signs alternate between positive and negative and no persistent pattern emerges. This indicates that the model residuals are essentially random in space, implying that no important spatial dependency has been omitted. Consequently, the conventional time‑series cross‑validation is sufficient for model evaluation, and explicit spatial cross‑validation is not required. This analysis provides strong evidence for the spatial validity of the model.

Table A3. Spatial autocorrelation of residuals (Moran’s I)

| **Year** | **agricultural_residue** | | **forestry_residue** | | **energy_crop** | | **animal_manure** | | **waste_sludge** | |
| --- | --- | --- | --- | --- | --- | --- | --- | --- | --- | --- |
|  | **Moran_I** | **p_value** | **Moran_I** | **p_value** | **Moran_I** | **p_value** | **Moran_I** | **p_value** | **Moran_I** | **p_value** |
| 2001 | -0.01 | 0.82 | 0.45 | 0.00 | -0.17 | 0.17 | -0.06 | 0.81 | 0.14 | 0.09 |
| 2002 | 0.19 | 0.03 | 0.23 | 0.02 | -0.21 | 0.09 | -0.05 | 0.84 | -0.18 | 0.14 |
| 2003 | 0.10 | 0.20 | 0.19 | 0.04 | -0.22 | 0.07 | 0.04 | 0.50 | -0.08 | 0.64 |
| 2004 | 0.00 | 0.71 | -0.16 | 0.30 | -0.08 | 0.62 | 0.18 | 0.04 | -0.12 | 0.41 |
| 2005 | 0.00 | 0.75 | -0.15 | 0.33 | 0.16 | 0.07 | 0.02 | 0.60 | -0.06 | 0.80 |
| 2006 | 0.04 | 0.49 | 0.08 | 0.33 | 0.07 | 0.32 | -0.02 | 0.88 | -0.03 | 0.94 |
| 2007 | -0.06 | 0.78 | 0.02 | 0.61 | -0.03 | 1.00 | 0.13 | 0.11 | -0.08 | 0.67 |
| 2008 | 0.15 | 0.08 | 0.11 | 0.15 | -0.13 | 0.34 | 0.09 | 0.24 | -0.17 | 0.17 |
| 2009 | -0.19 | 0.13 | 0.12 | 0.14 | -0.22 | 0.07 | 0.22 | 0.01 | -0.14 | 0.32 |
| 2010 | 0.00 | 0.74 | 0.03 | 0.53 | -0.23 | 0.06 | -0.11 | 0.48 | -0.09 | 0.57 |
| 2011 | 0.37 | 0.00 | -0.03 | 1.00 | 0.10 | 0.19 | 0.03 | 0.55 | 0.04 | 0.49 |
| 2012 | -0.10 | 0.52 | -0.07 | 0.74 | -0.02 | 0.88 | 0.02 | 0.62 | -0.22 | 0.07 |
| 2013 | -0.16 | 0.22 | -0.09 | 0.58 | -0.11 | 0.47 | 0.03 | 0.53 | -0.02 | 0.88 |
| 2014 | -0.19 | 0.13 | 0.08 | 0.32 | -0.21 | 0.09 | 0.07 | 0.32 | 0.23 | 0.01 |
| 2015 | 0.14 | 0.10 | -0.06 | 0.81 | -0.17 | 0.18 | -0.09 | 0.55 | 0.08 | 0.26 |
| 2016 | -0.06 | 0.78 | -0.11 | 0.60 | -0.04 | 0.98 | -0.08 | 0.63 | -0.05 | 0.84 |
| 2017 | 0.14 | 0.09 | 0.12 | 0.20 | -0.09 | 0.60 | -0.10 | 0.50 | -0.20 | 0.11 |
| 2018 | -0.31 | 0.01 | 0.17 | 0.09 | 0.01 | 0.64 | -0.08 | 0.62 | 0.00 | 0.76 |
| 2019 | -0.22 | 0.07 | -0.24 | 0.10 | 0.07 | 0.32 | -0.17 | 0.20 | -0.10 | 0.54 |
| 2020 | -0.21 | 0.08 | 0.00 | 0.73 | 0.15 | 0.07 | -0.22 | 0.07 | -0.08 | 0.65 |

Figures A1a–A1e present the SHAP summary plots for the random forest models of the five biomass types. In each plot, the y‑axis lists the most influential features in descending order of mean absolute SHAP value, and the x‑axis shows the SHAP value (marginal contribution of each feature to the model output). Red color indicates a high feature value, while blue indicates a low feature value.

Figure A1a. SHAP summary plot for agricultural residue random forest model

Figure A1b. SHAP summary plot for forestry residue random forest model

Figure A1c. SHAP summary plot for energy crop random forest model

Figure A1d. SHAP summary plot for animal manure random forest model

Figure A1e. SHAP summary plot for municipal waste & sludge random forest model

## B. Workflow Overview

The workflow consists of five main steps. First, provincial‑level totals (from statistical yearbooks and literature) are prepared together with spatial layers (land use, NPP, population). All layers are reprojected to WGS84 and resampled to a consistent 1 km grid. Second, suitable land use masks are applied: cropland for agricultural residues, forest land for forestry residues, marginal land for energy crops, while rural population maps are used for animal manure and urban population maps for municipal waste. Third, allocation weights are calculated as the product of the proxy variable (NPP for residues, population for manure/waste) and the corresponding mask. Fourth, weights are normalized by the provincial sum. Fifth, the provincial total is multiplied by the normalized weight to obtain the grid‑cell value. The downscaling operations were implemented in Python 3.10 using the rasterio and numpy libraries.

Figure B1. Workflow of spatial downscaling of provincial biomass potentials to 1 km grid cells.

The workflow is divided into a training phase (left) and a prediction phase (right). In the training phase, historical panel data (2001–2020, 31 provinces, 620 samples) undergo feature engineering, followed by feature selection using a preliminary random forest (retaining the most important features). A 5‑fold time‑series cross‑validation is then applied to evaluate model performance (RMSE, MAE, R²). Hyperparameters are optimized via grid search, resulting in the final random forest configuration: n_estimators = 150, max_depth = 6, min_samples_leaf = 15, min_samples_split = 20. Uncertainty is quantified as the standard deviation of predictions across all trees, and SHAP analysis is performed to interpret feature contributions. In the prediction phase, future feature variables (2021–2050) under the SSP2 socioeconomic pathway are fed into the trained model to obtain 2050 biomass potentials. All modeling was implemented in Python

using shap and scikit‑learn.

Figure B2. Workflow of random forest modeling and future biomass potential prediction.

## C. Key parameters for data assessment and prediction

Table C1. Summary of input datasets

| **Dataset** | **Resolution** | **Temporal coverage** | **Missing %** | **Handling method** |
| --- | --- | --- | --- | --- |
| Land use (RESDC) | 1 km | 2020 | 0% | Direct use |
| WorldPop population | 100 m → aggregated to 1 km | 2001-2020 | <1% | Interpolation + spatial allocation |
| Population historical | Provincial | 2001-2020 | 0% | Direct use |
| Population forecast (SSP2) | Provincial | 2021-2050 | 0% | Direct use |
| GDP development level | Provincial | 2001-2020 | <2% | Linear interpolation |
| Second industry added value | Provincial | 2001-2020 | <2% | Linear interpolation |
| Urbanization rate | Provincial | 2001-2020 | ~5% (early years) | Linear interpolation + forward/backward fill |
| Waste treatment rate | Provincial | 2001-2020 | ~3% | Linear interpolation |
| Straw collection coefficient | Provincial | 2001-2020 | 0% | Linear interpolation |
| Manure collection rate | Provincial | 2001-2020 | 0% | Linear interpolation |
| Population density | Provincial | 2001-2020 | <1% | Interpolation |
| SSP2 land use | 0.25° → aggregated to 1 km | 2021-2050 | 0% | Direct use |

Table C2. Input variables for future projections (2021–2050)

| **Input variable** | **Spatial resolution** | **Temporal frequency** | **Assumptions** |
| --- | --- | --- | --- |
| Population | Provincial | Annual (2021-2050) | Linear extrapolation of historical trend (2001-2020); no abrupt policy changes |
| GDP | Provincial | Annual | Linear extrapolation; last 2-year growth rate extended to 2050 |
| Cropland area | Provincial | Annual | Trend extrapolation based on recent 5 years, with national redline constraint (1.865 M ha) |
| Forest area | Provincial | Annual | Trend extrapolation based on recent 5 years |
| Marginal land area | Provincial | Annual | Trend extrapolation based on recent 5 years |
| Technology index | Provincial (calculated) | Annual | Sigmoid growth: 1/(1+e−0.1×(year−2015))1/(1+*e*−0.1×(year−2015)) |
| Technology growth rate (agriculture) | Provincial (constant) | Annual (2021-2030/2040/2050) | 0.8%, 1.2%, 1.5% respectively |
| Technology growth rate (forestry) | Provincial (constant) | Annual (2021-2030/2040/2050) | 0.3%, 0.4%, 0.5% respectively |
| Technology growth rate (energy crop) | Provincial (constant) | Annual (2021-2030/2040/2050) | 0.5%, 1.0%, 1.5% respectively |
| Straw collection coefficient | Provincial | Annual | Linear extrapolation of historical trend |
| Manure collection rate | Provincial | Annual | Linear extrapolation of historical trend |
| Waste treatment rate | Provincial | Annual | Linear extrapolation of historical trend |
| SSP2 land use / population | 1 km grid (aggregated to province) | Annual | SSP2 “middle-of-the-road” socioeconomic pathway |

## D. Assessment and predicted values of the calorific potential of five types of biomass resources

Table D1. 2020 Biomass Resource Assessment Calorific Potential (Unit PJ)

| **Province** | **agricultural residues** | **forestry residues** | **energy crops** | **animal manure** | **municipal waste** |
| --- | --- | --- | --- | --- | --- |
| **Anhui** | 542.05 | 349.70 | 97.67 | 218.92 | 30.49 |
| **Beijing** | 5.17 | 12.54 | 18.32 | 3.41 | 36.32 |
| **Fujian** | 60.26 | 567.74 | 235.43 | 173.37 | 38.43 |
| **Gansu** | 187.78 | 4.98 | 218.42 | 172.08 | 7.81 |
| **Guangdong** | 199.05 | 624.95 | 142.70 | 252.59 | 116.50 |
| **Guangxi** | 358.93 | 2139.48 | 577.06 | 256.82 | 15.08 |
| **Guizhou** | 148.86 | 179.25 | 561.56 | 140.66 | 11.96 |
| **Hainan** | 19.61 | 128.18 | 26.35 | 39.79 | 8.37 |
| **Hebei** | 608.73 | 62.35 | 105.18 | 239.34 | 34.99 |
| **Henan** | 1099.55 | 147.72 | 49.95 | 346.61 | 32.62 |
| **Heilongjiang** | 1108.93 | 68.06 | 26.59 | 171.98 | 10.55 |
| **Hubei** | 408.97 | 136.79 | 570.57 | 176.32 | 30.82 |
| **Hunan** | 413.47 | 270.90 | 474.74 | 235.20 | 25.18 |
| **Jilin** | 652.21 | 123.74 | 71.39 | 147.77 | 16.25 |
| **Jiangsu** | 465.51 | 118.34 | 25.00 | 142.62 | 92.16 |
| **Jiangxi** | 272.09 | 226.89 | 338.63 | 157.48 | 17.70 |
| **Liaoning** | 410.07 | 64.76 | 82.70 | 235.91 | 26.08 |
| **Inner Mongolia** | 676.72 | 48.17 | 360.12 | 353.40 | 9.99 |
| **Ningxia** | 60.12 | 0.00 | 74.01 | 51.24 | 4.73 |
| **Qinghai** | 19.99 | 1.08 | 14.35 | 147.24 | 1.31 |
| **Shandong** | 871.77 | 278.44 | 71.98 | 501.67 | 89.69 |
| **Shanxi** | 235.82 | 15.01 | 366.07 | 87.40 | 12.64 |
| **Shaanxi** | 199.52 | 18.34 | 332.53 | 76.89 | 16.68 |
| **Shanghai** | 10.40 | 0.00 | 0.36 | 3.75 | 46.25 |
| **Sichuan** | 541.90 | 167.71 | 425.80 | 382.88 | 40.13 |
| **Tianjin** | 34.00 | 13.49 | 1.73 | 17.95 | 14.72 |
| **Tibet** | 17.77 | 1.74 | 224.82 | 130.25 | 0.83 |
| **Xinjiang** | 488.60 | 35.21 | 75.46 | 260.89 | 5.85 |
| **Yunnan** | 333.91 | 504.19 | 1019.37 | 277.58 | 16.62 |
| **Zhejiang** | 76.89 | 113.77 | 70.49 | 46.39 | 69.10 |
| **Chongqing** | 141.87 | 42.34 | 140.35 | 79.83 | 21.91 |

Table D2. 2050 Biomass Resource Predicted Calorific Value Potential (Unit PJ)

| **Province** | **agricultural residues** | **forestry residues** | **energy crops** | **animal manure** | **municipal waste** |
| --- | --- | --- | --- | --- | --- |
| **Anhui** | 856.92 | 518.50 | 128.05 | 362.87 | 36.88 |
| **Beijing** | 10.35 | 24.89 | 27.39 | 9.26 | 37.53 |
| **Fujian** | 93.07 | 546.50 | 371.32 | 367.38 | 38.28 |
| **Gansu** | 288.35 | 8.01 | 322.35 | 324.45 | 7.80 |
| **Guangdong** | 287.81 | 2494.87 | 207.42 | 323.34 | 75.03 |
| **Guangxi** | 512.52 | 2734.58 | 777.37 | 415.33 | 14.68 |
| **Guizhou** | 226.87 | 323.52 | 772.92 | 305.45 | 14.20 |
| **Hainan** | 58.38 | 233.66 | 33.74 | 52.39 | 8.30 |
| **Hebei** | 899.03 | 114.19 | 142.08 | 323.00 | 39.18 |
| **Henan** | 1529.89 | 270.34 | 67.86 | 522.86 | 54.32 |
| **Heilongjiang** | 1538.22 | 116.09 | 33.55 | 367.02 | 13.93 |
| **Hubei** | 643.64 | 263.73 | 738.71 | 313.84 | 54.56 |
| **Hunan** | 633.73 | 400.88 | 618.18 | 362.87 | 25.11 |
| **Jilin** | 1147.43 | 225.56 | 96.92 | 328.76 | 20.29 |
| **Jiangsu** | 726.80 | 234.74 | 34.20 | 281.92 | 74.98 |
| **Jiangxi** | 420.59 | 316.94 | 469.24 | 314.06 | 17.56 |
| **Liaoning** | 663.10 | 123.32 | 104.95 | 415.26 | 32.17 |
| **Inner Mongolia** | 1285.81 | 83.58 | 484.21 | 557.78 | 13.95 |
| **Ningxia** | 84.40 | 0.05 | 100.46 | 52.39 | 4.59 |
| **Qinghai** | 30.25 | 1.39 | 25.85 | 212.99 | 1.30 |
| **Shandong** | 1438.22 | 430.36 | 96.09 | 531.25 | 75.05 |
| **Shanxi** | 383.90 | 29.61 | 498.38 | 94.73 | 15.24 |
| **Shaanxi** | 292.14 | 21.66 | 474.91 | 96.00 | 20.77 |
| **Shanghai** | 13.94 | 0.04 | 0.52 | 5.15 | 49.77 |
| **Sichuan** | 925.58 | 217.95 | 636.54 | 558.80 | 61.35 |
| **Tianjin** | 47.53 | 26.79 | 2.46 | 12.26 | 14.48 |
| **Tibet** | 27.10 | 8.45 | 327.25 | 209.28 | 0.84 |
| **Xinjiang** | 776.20 | 80.53 | 103.87 | 351.04 | 5.65 |
| **Yunnan** | 533.12 | 2693.04 | 1370.45 | 415.47 | 16.50 |
| **Zhejiang** | 112.45 | 112.28 | 97.49 | 54.89 | 68.36 |
| **Chongqing** | 207.07 | 79.84 | 193.53 | 96.75 | 24.17 |
